# Development and evaluation of a pediatric hospital medicine board review course

**DOI:** 10.1186/s12909-022-03862-1

**Published:** 2022-11-19

**Authors:** Lisa E. Herrmann, Yemisi O. Jones, Benjamin Kinnear, Amy Rule, Laura Piper, Samir S. Shah, Melissa Klein

**Affiliations:** 1 Department of Pediatrics, University of Cincinnati College of Medicine, Cincinnati, OH USA; 2 Division of Hospital Medicine, Cincinnati Children’s Hospital Medical Center, Cincinnati, OH USA; 3 Division of General Pediatrics, Cincinnati Children’s Hospital Medical Center, Cincinnati, OH USA

**Keywords:** Program evaluation, Continuing medical education, Board review course, Pediatric hospital medicine

## Abstract

**Background:**

The American Board of Medical Specialties recognized Pediatric Hospital Medicine (PHM) for subspecialty certification in 2016, with the first certification exam in 2019. To address the need for exam preparatory materials, we designed and evaluated a novel PHM board review course that was offered both in-person and online.

**Methods:**

Course content was based on the American Board of Pediatrics (ABP) PHM certifying exam outline. Course objectives were developed from published PHM core competencies and the 2012 ABP general pediatrics content objectives. National experts served as faculty, presenting didactic sessions, and contributing to a question bank for high-yield review. For program evaluation, we applied the Kirkpatrick Model, evaluating estimated exam pass rates (Level 4), participant learning (Level 2) via post-presentation practice questions, and participants’ ratings of presenters (via five-point Likert scale) and satisfaction (Level 1).

**Results:**

There were 112 in-person and 144 online participants with estimated pass rates of 89 and 93%, respectively. The mean correct response for the post-presentation knowledge questions was 84%. Faculty effectiveness ratings ranged from 3.81 to 4.96 (median score 4.60). Strengths included the pace of the course, question bank, and printed syllabus. Suggestions for improvement included question bank expansion, focus on “testable” points rather than general information, and challenges with long days of didactic presentations.

**Conclusions:**

This novel PHM board review course demonstrated effectiveness. Hospitalists preferred focused “testable” information, an active learning environment, and a robust question bank. Future preparatory courses should consider including more opportunities for practice questions, focused content review, and learner engagement.

**Supplementary Information:**

The online version contains supplementary material available at 10.1186/s12909-022-03862-1.

## Background

The American Board of Medical Specialties officially recognized Pediatric Hospital Medicine (PHM) for subspecialty certification in 2016 [1]. PHM is an evolving field, reflective of the growing expertise of hospital-based pediatricians caring for acutely ill children and an increasingly complex, diverse patient population [2]. Physicians seeking PHM board certification now must pass a standardized examination. To be eligible to sit for this board exam, hospitalists must have either completed a PHM fellowship or be several years into practice [3].

Board certification can establish professional identity, update knowledge, improve patient care [4], and may be a requirement for employment [5]. Physicians spend substantial preparation time and commit large sums of money for test fees ($2900 for the PHM exam) [6] and preparatory materials. In addition to necessity and cost, the stakes are further raised since the PHM exam is only offered every other year, limiting exam-taking and remediation opportunities. Despite the high stakes, few PHM-specific study resources existed at the time of the first exam, and none had reported efficacy.

The lack of PHM certification exam preparatory materials created the need for a board review course. Although initial board certification courses for other exams exist, these are usually taken by junior physicians soon after training. Optimal methods to prepare practicing hospitalists for licensing exams have not been explored. Many individuals taking the first PHM board exam had been out of training and in practice for several years, thus removed from the frequent standardized tests associated with medical training. These adult learners may have different learning needs and expectations than test-takers who recently completed training. Course content and educational strategies needed to be based on adult learning principles to optimize knowledge retention and recall. Adult learners are self-directed, draw from prior experiences, focus on content with immediate relevance and impact on their professional lives, and approach learning through problem-solving rather than memorization [7].

To address these educational gaps, we designed and evaluated a board review course for the 2019 PHM board exam.

## Methods

This paper describes the development and program evaluation of our PHM board review course. We used Kern’s six-step approach to curriculum development [8].

### Global and targeted needs assessments (Kern’s steps 1 and 2)

The American Board of Pediatrics (ABP) developed and published a PHM board exam content outline, consisting of 13 knowledge domains, with associated subdomains and examination weights (i.e., the percentage of exam questions assigned to each area). Each exam question was designed to address a “core task” that reflects how medical knowledge can be applied in clinical practice. Core tasks included core science and pathophysiology, epidemiology and risk assessment, diagnosis and testing, and management and treatment [9]. While the domains, subdomains, diagnoses, and core tasks outlined in the ABP content outline provided some guidance for studying, no discrete learning objectives existed to guide exam preparation [10].

### Goals and objectives (Kern’s step 3)

Two course directors developed the course in consultation with the PHM division director and a planning committee of practicing hospitalists who helped develop content and a course question bank. Divisional administrative assistants and institutional Continuing Medical Education (CME) staff provided planning support. With a global goal of preparing learners to pass the PHM board exam, we created and adapted individual objectives for each domain and subdomain on the ABP content outline. We modified course content objectives from a set of previously published PHM core competencies [11], as well as related 2012 ABP general pediatrics content objectives [12]. The board review course co-directors, who have formal medical education training, reviewed and revised objectives (632 total, average 19 per session with a range of 4–41) via an iterative process with consensus-building.

### Educational strategies and implementation (Kern’s steps 4 and 5)

We developed the course utilizing adult learning principles to enhance learning and facilitate recall for the exam. Specifically, we acknowledged that adult learners focus on content with immediate relevance and impact on professional lives, draw from prior clinical experiences, are self-directed, and approach learning through problem-solving, rather than memorization [7]. The course occurred approximately 2 months prior to the exam, so it was important that studying initiated during the course continue with a high degree of retention. In accordance with andragogy [13], our learners were motivated given the important implications of board certification. At the start of the course, we described the source of the content objectives, demonstrating the relevance to both clinical practice and exam content. Self-directed learning was incorporated into the in-person course, allowing participants to identify content relevant to their learning needs based on prior experiences and clinical exposure. In addition, development of a course question bank and a test-taking strategies presentation by a medical education learning specialist promoted use of practice questions as a study technique.

We invited national PHM experts to serve as course faculty and provided them with content objectives and suggested time to devote to each content area, based on the examination weight on the ABP content outline. We requested that each speaker develop a presentation covering their assigned objectives, focusing on high-yield clinical content. Each didactic session was designed to provide a focused review; we encouraged use of tables and other visuals to aid studying. Faculty also created five board-style multiple choice questions on their assigned topic to contribute to a course question bank. Course co-directors and the question bank editor reviewed presentations and questions, and faculty revised both based on feedback.

The in-person course was delivered over three full days (24.5 hours, Supplement 1). Morning content was presented in a didactic format focused on content objectives. Session length averaged 30 minutes with five-minute breaks between sessions to allow for questions and speaker transitions. Longer mid-morning, mid-afternoon, and lunch breaks were scheduled. Course participants received binders with copies of slides, notes pages, and supplemental material to complement the presentations. All sessions were recorded, and videos and slides were available for review after the course via an online portal.

Afternoon sessions consisted of hands-on or self-directed learning targeted toward procedural skills (e.g., procedural knowledge, neonatal resuscitation) and non-clinical, non-technical skills (e.g., research methods, education, quality improvement, safety, leadership). Recognizing that course participants had unique prior experiences and knowledge based on their job, role, and practice setting, the afternoon format allowed participants to choose sessions of highest yield based on their individual learning needs. For sessions focused on procedural skills, stations with simulation trainers were available along with checklists with indications, contraindications, required supplies, steps for procedure completion, and potential complications. To promote recall and retention, we provided participants with individual checklists containing blanks in key sections to complete as they moved through the stations.

A medical education learning specialist from our institution’s affiliated medical school presented a test-taking strategy session. Since many course participants were more removed from high-stakes exams and needed to juggle work and family responsibilities with study time, test anxiety was high. This session included evidence-based study strategies (e.g., active learning, spaced repetition, concept mapping) and techniques for reasoning through multiple-choice questions, with application of these techniques to a set of practice questions reviewed as a group. The learning specialist also provided general tips for test-taking, such as sleep hygiene, nutrition, and managing test anxiety.

Course faculty questions were collated into a question bank with over 150 questions and explanations for correct answers. After review by the question bank editor, we uploaded questions to an online platform with quiz creation capabilities. In addition to traditional multiple-choice questions, we created a visual diagnosis section, which included photographs of physical exam findings with associated diagnostic or management questions. We displayed these visual diagnosis questions in the conference hall for review during course breaks and later in the online portal.

We made recordings of didactics, the course syllabus, presentation slides with notes pages, and access to the question bank available online via our institution’s CME web portal to be accessed afterwards via a desktop or mobile device. Individuals who were unable to attend the in-person course could purchase an electronic-only version of the course materials for self-study. We offered CME hours and Part 2 Maintenance of Certification (MOC) [14] for both in-person and online course participants.

### Program evaluation (Kern’s step 6)

We applied Kirkpatrick’s Model (KM), a best practice for analyzing impact of a training program, to our program evaluation. KM evaluates curricula by measuring multi-level outcomes: 1) participant reactions, 2) learning, 3) behavior change, and 4) desired results of the program or organizational change [15, 16]. We estimated a pass rate for course attendees by cross-referencing individuals registered for the course with the publicly available roster of diplomates on the ABP website (Level 4). At the completion of each topic session during the in-person course, the speaker displayed a board-style question via an audience response system (ARS) to provide an opportunity for participants to test their knowledge acquisition as a formative measure (Level 2). Course evaluations (Supplement 2), based on standard questionnaires utilized by our institution’s CME office and reviewed by educators and hospitalists for content validity, assessed participant reactions and satisfaction (Level 1). Immediate post-course evaluations assessed the effectiveness of content delivery, whether the course met learning needs, and sought feedback for future course iterations. These questions were based on a seven-point Likert scale (1 = strongly disagree, 7 = strongly agree). We sent a delayed post-exam evaluation to both in-person and online course participants 4 months after the in-person course, 2 months after the exam. Questions were based on a scale of 1–100 assessing agreement with specific statements about the course. Both evaluations contained open-ended questions allowing respondents to identify areas of strength and for improvement for the course. The course directors reviewed free response comments to identify themes in responses. In-person course participants also rated the effectiveness of each faculty presenter on a five-point Likert scale (1 = strongly disagree with statement of effectiveness, 5 = strongly agree) at the completion of each didactic session using an ARS.

## Results

The in-person course had 112 attendees representing 28 states and the District of Columbia. The online course had 144 registrants representing 36 states, the District of Columbia, and Puerto Rico. Most course participants had been practicing at least 4 years, 97 and 98% for the in-person and online courses, respectively; the remainder were PHM fellows. We calculated a minimum pass rate of 89% for the in-person course and 93% for individuals with online access only (assuming all participants took the exam in 2019), compared with a national pass rate of 84% [17]. For sessions in which the ARS was used (26 out of 31 sessions), the mean correct response for the post-presentation knowledge question was 84%.

Eighty-seven of 112 (78%) in-person participants and 63 of 144 (44%) online participants completed the immediate post-course evaluation (Table 1). Identified areas of strength included presentations from experts in the field, the overall course pacing, the test-taking strategies session, and question bank. Suggestions for improvement highlighted a need for further standardization of presentations and provision of a best practices guide for speakers on how to develop and deliver a presentation more focused on “testable” points rather than general clinical information. In addition, participants suggested the question bank have more robust rationale for correct versus incorrect answers. Many in-person participants commented on the challenges of prolonged sitting for didactics and limited interactivity during the morning content-heavy sessions. Faculty effectiveness ratings ranged from 3.81 to 4.96 (out of 5) with a median score of 4.60.

**Table 1:** Immediate post-course evaluation for both the in-person and online PHM board review course

	In-person course *(n = 112)* 87 responses (78%)	Online-only access *(n = 144)* 63 responses (44%)
**Evaluation Statement**	**Mean score**^a^	**Mean score**^a^
I would recommend this learning experience to a colleague.	6.65	6.02
The format of this learning experience was appropriate for its content	6.50	–
The material presented during this learning experience met my learning needs.	6.47	5.92
I will be able to use what I gained from this learning experience to improve my clinical skills.	6.26	–
I will be able to use what I learned in this learning experience to improve my patients’ medical or quality of life outcomes.	6.09	6.00
This activity will positively impact the clinical skills and performance of my healthcare team.	–	5.95

^a^Scoring based on 7-point Likert Scale (1 = strongly disagree, 7 = strongly agree)

Forty in-person course participants (36%) and 34 online participants (24%) responded to the delayed post-exam evaluation (Table 2). Respondents from both groups reported the learning format was appropriate for its content and the learning experience adequately prepared the individual for the PHM board exam. Respondents rated the most beneficial components of the course to be the lectures, the question bank, and the printed syllabus with content objectives and lecture notes. There was a preference for content delivered by pediatric hospitalists as opposed to specialists, with the rationale that hospitalists were better able to identify information pertinent to hospitalist practice and to the field. Many individuals requested an expanded question bank with more practice questions and more detailed descriptions of response rationale. Individuals with online-only access commented that the online platform was cumbersome and difficult to navigate. Overwhelming positive feedback on the test-taking strategies session was consistent across both formats.

**Table 2:** Post-exam evaluation that occurred 2 months post-PHM Board exam and 4 months post-PHM board review course

	In-person course *(n = 112)* 40 responses (36%)	Online-only access *(n = 144)* 34 responses (24%)
**Evaluation Statement**	**Mean score**^a^	**Mean score**^a^
I would recommend this learning experience to a colleague.	91	83
The format of this learning experience was appropriate for its content.	90	83
The material presented during this learning experience met my needs.	86	80
This learning experience adequately prepared me to sit for the PHM board exam	84	76
**Course’s most beneficial learning strategies**	**Number selected**^b^	**Number selected**^b^
In-person lectures	38 (95%)	n/a
Question bank	29 (72.5%)	22 (68.8%)
Printed course syllabus (content objectives and lecture slides)	26 (65%)	n/a
Visual diagnosis questions	24 (60%)	15 (46.9%)
Recorded videos of didactics	21 (52.5%)	28 (87.5%)
Online course syllabus	20 (50%)	25 (78.1%)
Procedure checklists	8 (20%)	6 (18.8%)
Procedure/Simulation break-out sessions	5 (12.5%)	n/a

^a^Scoring based on scale of 0–100 assessing agreement with statement

^b^Participants could select more than one response option

## Discussion

Our PHM board review course was positively reviewed by both in-person and online participants. In-person or recorded didactics, the course syllabus with content objectives and lecture slides, and the question bank received the highest ratings as teaching strategies, and participants demonstrated a high examination pass rate. Based on our experience developing a board review course for the first offering of a new subspecialty board certification exam, we reflect on lessons learned that may inform future exam preparatory courses for practicing physicians.

Post-course and post-exam evaluations highlighted participants’ expectation for content to be focused on testable information, as opposed to more generalized clinical overviews. Many of our presentations incorporated a general overview, as is common in undergraduate, graduate, and continuing medical education, rather than focusing on *application* of knowledge to diagnosis and treatment of the conditions on the content outline. The adult learners in our course requested immediate relevance of the content to their learning goals [13], in this case passing the board exam. Future iterations of the course should focus on high-yield, practical information that could appear on the exam or in clinical scenarios.

In-person course participants noted that they struggled with the monotony of so many didactic presentations. While we attempted to infuse interactivity via practice questions with an ARS at the end of each presentation and hands-on procedural sessions, we were limited by the vast amount of information to cover in a short period of time. More traditional didactics or lectures may be an appropriate educational strategy when the primary goal is cognitive/information transfer or when a large amount of information is needed by a larger group of learners in a short period of time [18]. However, participants requested more active learning and engagement with the material. Active learning techniques can enhance learner engagement and retention by promoting application of the material while it is being presented [19]. Methods to achieve a more active learning environment within the constraints of presenting a large amount of information in limited time could include the use of clinical cases to guide presentation of information, interspersed practice questions, and increased use of an ARS for polling participants [20]. Our participants reported a preference for practice questions as a study method; thus, a more robust question bank with detailed explanations, combined with questions infused throughout course presentations could improve this course.

Our program evaluation did have some limitations. First, surveys to evaluate the course were not standardized between in-person and online formats, making results difficult to compare. Second, the delayed post-exam evaluations had lower response rates, which may introduce nonresponse bias. Third, we did not track how online-only participants interacted with the course material (e.g., number of didactics watched, questions completed), which may explain the lower response rates and overall lower evaluation scores. Fourth, our interpretation of learner knowledge acquisition via the post-presentation questions is limited as we did not include a pre-course knowledge assessment for comparison; however, these questions were intended as a formative measure for course participants to direct their own learning after the course. Fifth, we could only estimate pass rates as we did not ask participants if they sat for the 2019 exam, which would have allowed for a more accurate denominator for our pass rate calculation. However, if fewer course participants took the 2019 board exam, our pass rate would be higher than currently estimated. Additionally, attempts to determine statistical significance of board exam pass rates was challenging as course participants were included in both course participant pass rates and the national pass rate, confounding any potential analysis. We also did not assess all Kirkpatrick’s levels as assessing behavior change (Level 3) of all participants was not feasible and clinical performance was not a primary objective of this study. Finally, as we did not prospectively collect demographic data, participants in our PHM board review course may not be representative of all practicing pediatric hospitalists.

As we prepare for the next iteration of the PHM board review course amidst a global pandemic that necessitates a fully virtual course, we have been struck by the importance of human interaction that transcends the personal into our professional lives [21]. It is through this lens that we have begun incorporating the evaluations and feedback from the 2019 course. The challenge of redesigning the course given pandemic limitations has afforded us the opportunity to enhance learning and retention in a way that was not possible with the traditional didactic-focused format. We recognize that the flexibility of virtual asynchronous learning may remove the time limitations we experienced during the inaugural course and creates opportunities for innovative methods of bringing people together, such as development of communities of practice (CoP), informal learning communities that can enhance learning among individuals in a social environment. CoP were initially described in education and business literature and have been increasingly explored in the health professions [22]. Board review courses, such as the one we describe here, can serve as a stimulus for the creation of CoP focused on board exam preparation. This may include the creation of informal study groups, blogs, or wikis for participants to post about their experience studying for the exam and seek feedback from other course participants [23], or even live chats from content experts. In addition, participants will be able to view shorter learning segments over a longer time period, providing the opportunity to reinforce knowledge to clinical practice and review via questions.

## Conclusion

Based on our results, we recommend that future medical board exam courses include focused review of content relevant to exam and practice, ample opportunities for learner engagement, and robust practice questions. For practicing physicians who may not have a ready learning community, making connections for accountability and motivation may provide an added benefit.

## Supplementary Information

**Additional file 1.** Course Schedule, Pediatric Hospital Medicine Board Review Course Schedule, This table presents the course schedule, including topics, content delivery method, and number of content objectives address.

**Additional file 2.** Evaluation Tools, Evaluation tools, Questions asked on the immediate post-course evaluations and the post-exam evaluations.

## Data Availability

The datasets during and/or analyzed during the current study available from the corresponding author on reasonable request.

## Abbreviations

PHM: Pediatric Hospital Medicine
ABP: American Board of Pediatrics
CME: Continuing Medical Education
MOC: Maintenance of Certification
KM: Kirkpatrick Model
ARS: Audience response system
CoP: Communities of Practice

## References

[1] American Board of Medical Specialties. American Board of Medical Specialties Officially Recognizes Pediatric Hospital Medicine Subspecialty Certification [Press Release]. Retrieved 29 October 2020. https://www.abms.org/media/120095/abms-recognizes-pediatric-hospital-medicine-as-a-subspecialty.pdf.

[2] Burns KH, Casey PH, Lyle RE, Mac Bird T, Fussell JJ, Robbins JM (2010). Increasing prevalence of medically complex children in US hospitals. Pediatrics.

[3] American Board of Pediatrics. Pediatric Hospital Medicine Certification 2019. Retrived 29 October 2020. https://www.abp.org/content/pediatric-hospital-medicine-certification#training.

[4] Lipner RS, Hess BJ, Phillips RL (2013). Specialty board certification in the United States: issues and evidence. J Contin Educ Heal Prof.

[5] Freed GL, Uren RL, Hudson EJ, Lakhani I, Wheeler JR, Stockman JA (2006). Policies and practices related to the role of board certification and recertification of pediatricians in hospital privileging. JAMA.

[6] American Board of Pediatrics. Exam Dates and Fees for Subspecialties 2020. Retrieved 23 December 2020. https://www.abp.org/content/exam-dates-and-fees-subspecialties.

[7] Shannon S (2003). Adult learning and CME. Lancet.

[8] Kern DE, Thomas PE, Hughes ME (2009). Curriculum development for medical education: a six-step approach.

[9] Mittal V, Shah N, Dwyer AC, O’Toole JK, Percelay J, Carlson D, et al. Developing content for pediatric hospital medicine certification examination using practice analysis. Pediatrics. 2020;146(2). 10.1542/peds.2019-3186. 10.1542/peds.2019-3186 32727825

[10] American Board of Pediatrics. Pediatric Hospital Medicine Content Outline 2019. Retreived 2 April 2019. https://www.abp.org/sites/abp/files/pdf/hospital_medicine_content_outline.pdf.

[11] Stucky ER, Ottolini MC, Maniscalco J (2010). Pediatric hospital medicine core competencies: development and methodology. J Hosp Med.

[12] American Board of Pediatrics. Content Outline: General Pediatrics 2012. Retrieved 2 April 2019. https://www.abp.org/sites/abp/files/pdf/blueprint_gp_2016.pdf.

[13] Knowles M (1984). Andragogy in action: applying modern principles of adult learning.

[14] American Board of Pediatrics. Lifelong Learning and Self-Assessment (Part 2) 2020. Retreived 2 November 2020. https://www.abp.org/content/lifelong-learning-and-self-assessment-part-2.

[15] Kirkpatrick JD, Kirkpatrick WK (2016). Kirkpatrick's four levels of training evaluation.

[16] Li S-TT, Klein MD, Balmer DF, Gusic ME (2020). Scholarly evaluation of curricula and educational programs: using a systematic approach to produce publishable scholarship. Acad Pediatr.

[17] American Board of Pediatrics. Initial Certifying Examination First-Time Taker Passing Rates 2020. Retreived 13 November 2020. https://www.abp.org/sites/abp/files/pdf/exam-pass-rates-init-cert.pdf.

[18] Farrah SJ, Galbraith M (2004). Lecture. Adult learning methods: a guide for effective instruction.

[19] Van Amburgh JA, Devlin JW, Kirwin JL, Qualters DM. A tool for measuring active learning in the classroom. Am J Pharm Educ. 2007;71(5). 10.5688/ag710585. 10.5688/aj710585 PMC2064883 17998982

[20] Silberman M (2006). Active training: a handbook of techniques, designs, case examples, and tips.

[21] Houchens N, Tipirneni R (2020). Compassionate communication amid the COVID-19 pandemic. J Hosp Med.

[22] Li LC, Grimshaw JM, Nielsen C, Judd M, Coyte PC, Graham ID (2009). Evolution of Wenger's concept of community of practice. Implement Sci.

[23] Yang S-H (2009). Using blogs to enhance critical reflection and community of practice. Educ Technol Soc.

