# Supplementary material for: Development and evaluation of a pediatric hospital medicine board review course
Source: BMC Med Educ. 2022 Nov 19;22:804. doi: 10.1186/s12909-022-03862-1 (PMC9675241; doi:10.1186/s12909-022-03862-1)
Supplement: Supplementary file 1 — Additional file 1. Course Schedule, Pediatric Hospital Medicine Board Review Course Schedule, This table presents the course schedule, including topics, content delivery method, and number of content objectives address. [file 12909_2022_3862_MOESM1_ESM.pdf]

**Development and Evaluation of a Pediatric Hospital Medicine Board Review Course**

Lisa E. Herrmann, MD MEd; Yemisi O. Jones, MD MEd; Benjamin Kinnear, MD MEd; Amy Rule, MD MPH; Laura Piper, MD; Samir S. Shah, MD MSCE; Melissa Klein, MD MEd

Corresponding author: Lisa E. Herrmann, University of Cincinnati College of Medicine/Cincinnati Children's Hospital Medical Center; lisa.herrmann@cchmc.org

**Supplement.** Pediatric Hospital Medicine Board Review Course Schedule

| **Day 1** | | | **Content Objectives** |
|---|---|---|---|
| 9:45-9:55 | Course introduction/Orientation | | |
| 10:00-10:30 | Rheumatology/Vasculitis | Interactive didactic | 31 |
| 10:35-11:05 | Renal | Interactive didactic | 26 |
| 11:10-11:55 | Neurology | Interactive didactic | 41 |
| 12:00-12:45 | *LUNCH* – Test taking strategies | | n/a |
| 12:45-1:15 | Infant topics | Interactive didactic | 24 |
| 1:20-1:50 | Pulmonary 1 | Interactive didactic | 33 |
| 1:55-2:25 | Injuries and exposures | Interactive didactic | 21 |
| 2:25-2:35 | *BREAK** | | |
| 2:35-3:25 | Children with medical complexity | Interactive didactic | 23 |
| 3:25-3:40 | Medical devices and technology | Interactive didactic | 22 |
| 3:45-4:00 | Procedural sedation overview | Interactive didactic | 16 |
| 4:00-5:15 | Breakout sessions – Medical procedures and devices | Interactive session with trainers | n/a |
| | | | |
| **Day 2** | | | |
| 7:30-8:00 | *BREAKFAST* | | |
| 8:00-8:30 | Behavioral/Mental Health Conditions 1 | Interactive didactic | 8 |
| 8:35-9:05 | Inborn errors of metabolism | Interactive didactic | 20 |
| 9:10-9:40 | Gastrointestinal I | Interactive didactic | 23 |
| 9:45-10:00 | Dermatology | Interactive didactic | 9 |

| | | | |
|---|---|---|---|
| 10:00-10:10 | *BREAK** | | |
| 10:10-10:40 | Orthopedics/child maltreatment | Interactive didactic | 23 |
| 10:45-11:25 | Endocrine/metabolic | Interactive didactic | 19 |
| 11:30-12:15 | Head/neck infections and Fever of Unknown Origin (FUO) | Interactive didactic | 40 |
| 12:15-1:15 | *LUNCH* - Test taking strategies | | |
| 1:15-1:45 | Allergy/Immunology | Interactive didactic | 5 |
| 1:50-2:40 | Evidence-based high-value healthcare | Interactive didactic | 10 |
| 2:45-3:15 | Pulmonary 2 | Interactive didactic | 24 |
| 3:15-3:25 | *BREAK** | | |
| 3:25-4:25 | Newborn care | Interactive didactic | 15 |
| 4:25-4:40 | Introduction to NRP | Interactive didactic | 6 |
| 4:45-5:30 | Breakout sessions – Delivery room care/procedures | Interactive session with trainers | n/a |
| | | | |
| **Day 3** | | | |
| 7:30-8:00 | *BREAKFAST* | | |
| 8:00-9:00 | Cardiovascular | Interactive didactic | 38 |
| 9:05-9:35 | Behavioral/mental health conditions 2 | Interactive didactic | 9 |
| 9:40-10:10 | Hematology/Oncology | Interactive didactic | 31 |
| 10:10-10:25 | *BREAK** | | |
| 10:25-10:45 | Gynecology | Interactive didactic | 9 |
| 10:50-11:30 | Surgical co-management | Interactive didactic | 29 |
| 11:35-11:55 | Gastrointestinal 2 | Interactive didactic | 14 |
| 12:00-1:00 | *LUNCH* | | |
| 1:00-3:30 | <u>Self-directed breakout groups</u>:<br>Patient/family-centered care and care transitions<br>Ethics/Legal issues<br>Advocacy/Leadership<br>Quality Improvement/Patient Safety<br>Teaching and Education | Interactive didactics | <br>18<br><br>4<br>13<br>18 |

| | | | |
|---|---|---|---|
| | Research/Knowledge in scholarly activities | | 5<br>5 |
| 3:30-3:45 | Wrap-up/Course evaluations | | |

*Visual Diagnosis pictures and quizzes will be available during course breaks

Abbreviations: NRP – Neonatal Resuscitation Program
