# Supplementary material for: Development and evaluation of a pediatric hospital medicine board review course
Source: BMC Med Educ. 2022 Nov 19;22:804. doi: 10.1186/s12909-022-03862-1 (PMC9675241; doi:10.1186/s12909-022-03862-1)
Supplement: Supplementary file 2 — Additional file 2. Evaluation Tools, Evaluation tools, Questions asked on the immediate post-course evaluations and the post-exam evaluations. [file 12909_2022_3862_MOESM2_ESM.pdf]

Supplement 2. Evaluation tools

Post-course evaluation (live course)

Free response questions

1. What did you like best about this activity?
2. What did you like least about this activity?
3. Was there clinical content necessary for board preparation that was missing or underrepresented in this course? If YES, please explain.

Rate the degree to which you agree with the statement(s) below. (7 = Strongly Agree; 1 = Strongly Disagree)

4. I would recommend this learning experience to a colleague.
5. The format of this learning experience was appropriate for its content.
6. The material presented during this learning experience met my needs.
7. I will be able to use what I gained from this learning experience to improve my clinical skills.
8. I will be able to use what I gained from this learning experience to improve my patients' medical and/or quality of life outcomes.

Post-course evaluation (online course)

Rate the degree to which you agree with the statement(s) below. (7=Strongly Agree, 1=Strongly Disagree)

1. I would recommend this learning experience to a colleague.
2. The material presented during this learning experience meets my needs.
3. I will be able to use what I gained from this learning experience to improve my patients' medical and/or quality of life outcomes.
4. This activity will positively impact the clinical skills and performance of my healthcare team.

Post-exam evaluation (live and online courses)

Sliding scale 1-100 – please rate your level of agreement (1=disagree, 100=agree)

1. I would recommend this learning experience to a colleague.
2. The format of this learning experience was appropriate for its content.
3. The material presented during this learning experience met my needs.
4. This learning experience adequately prepared me to sit for the PHM board examination.

Check all that apply

5. I found the following resources helpful in preparing for the board examination (check all that apply):
    a. In-person lectures *(live course only)*

b. Videos of lectures
c. Printed syllabus *(live course only)*
d. Online syllabus
e. Procedure checklists *(live course only)*
f. Procedure breakout sessions *(live course only)*
g. Course question bank
h. Visual diagnosis
i. New England Journal of Medicine question bank *(live course only)*
j. Other (please specify)

Free response questions

6. What content was over-emphasized in the board review course?
7. What content could have been better represented in the board review course?
8. Do you have other feedback for the planners?
